# The effects of a designed program on oxygen saturation and heart rate of premature infants hospitalized in neonatal intensive care unit of Al-Zahra Hospital in Isfahan in 2008-2009

**Published:** 2010

**Authors:** Parvin Taheri, Eidan Abbasi, Zahra Abdeyazdan, Nahid Fathizadeh

**Affiliations:** * Department of Pediatric Nursing, School of Nursing and Midwifery, Isfahan University of Medical Sciences, Isfahan, Iran; ** School of Nursing and Midwifery, Isfahan University of Medical Sciences, Isfahan, Iran; *** Associate Professor, Nursing and Midwifery Cares Research Center, School of Nursing and Midwifery, Isfahan University of Medical Sciences, Isfahan, Iran; **** Department of Midwifery, School of Nursing and Midwifery, Isfahan University of Medical Sciences, Isfahan, Iran

**Keywords:** Programmed instruction, prematurity, newborn, pulse oximetry, noise, light

## Abstract

**BACKGROUND:**

Prematurity is the main cause of death in infants under one year of age and is the main reason for neonatal intensive care unit (NICU) hospitalization. The stressful environment of NICU exposes preterm infants to inappropriate stimuli. This study aimed to determine and compare the mean heart rate and oxygen saturation of premature infants before and during a designed program in NICU.

**METHODS:**

In a clinical trial study (before-after intervention) on a single group, 31 hospitalized premature newborns in NICU of Al-Zahra Hospital in Isfahan were selected by simple continuous sampling method. Data were collected through interview, observation and checklist records. The data were analyzed using SPSS and descriptive and inferential statistics.

**RESULTS:**

Out of 31 premature infants in the study, 60% were boys and 35% were girls. The mean (standard deviation) of oxygen saturation before and during the designed program were 92.80 (2.54) and 94.22 (2.59) percent, respectively. The results of paired t test showed a significant difference between the means of oxygen saturation of the infants before and during the program (p = 0.048), but there was no significant difference between the mean of the infants’ heart beat before and during the intervention.

**CONCLUSIONS:**

The findings showed that applying daily silence periods can greatly help to increase oxygen saturation and can improve the growth of premature infants. Therefore, by providing more facilities in clinical environments of NICU, conducting programs to reduce light and noise in these wards would be possible.

Some newborns are at a higher risk of mortality and are called high risk infants, because the gestational age or their birth weight put them at a higher-than-average risk of disease and death. Since most infants hospitalized in NICU are born preterm, the problems of high risk infants are mainly related to prematurity.^1^ Thirty eight percent of mortalities in the first 5 years of age belongs to prenatal period and out of these, 28% is related to preterm birth.^2^ The results of statistics in Iran show that in 1980, 13% of newborn were preterm, while in 2006 more than 30% of births were preterm.^3^

In spite of constant progress in maternity and prenatal health care which has led to improvement of pregnancy results, the problem of preterm birth still exists. Premature infants are usually underweight because they spend a shorter time in uterus.^4^ Survival rate of preterm infants is associated with birth weight and the lower the birth weight is, the higher the mortality rate is.^1^ With birth, the newborn enters a world with lots of light, loud and unexpected noises, where the temperature around it changes fast. In this environment, there are no uterine movements. In addition, the infant is exposed to painful experiences.^4^

Transition to extra uterine life is a major stress for all newborns. In this period, the internal rhythms, physiological functions and behavioral characteristics that have been organized before birth to adopt with uterine life should be organized to adopt with life out of uterus. A preterm infant experiences more problems due to prematurity of various systems of body especially central nervous system in creating the new organization.^5^ Premature infants often need intensive care to survive. NICU environment is a potential source of stress for infants because premature infants are exposed to continuous bombardment of inappropriate stimuli there.^4^ One of the unusual stimuli experienced by infants in this environment is noises.^5^ A study by Slevin et al showed that designing a care environment by reducing noise and light, unit staff activities and transferring leads to a decrease in diastolic blood pressure and mean arterial blood pressure as well as infants’ movement activities which are expression of their less stress as a result of improved care environment.^6^

Pillitteri believes that the amount of rest and stimuli needed for premature infants is not clear and NICU is anyway different from the environment the baby experiences in uterine environment. One of the unusual stimulants that infants experience in NICU is noise.^5^

Brandon et al also showed that providing an environment with periods of light and noise reduction leads to increasing infants’ sleep time and, improvement of nutrition and increasing their weights.^7^

Despite all its advantages, NICU has some harmful effects as well. Environmental conditions affect the baby’s physiological status. The heart rate, respiratory rate and oxygen consumption can increase in newborns in response to environmental events such as loud closure of a door, suction, telephone and alarm sounds, bathing or even approach of a care provider.

However, in our hospital there has been no effort to revise the patterns of physical and social stimulants in routine cares.^4^ Considering the above points and the effects of a safe environment on premature infants’ physiologic activities, which leads to their growth, the researcher wonders why there is no designed plan to include calm periods with reducing sounds and lights in NICUs in Iran. Since there was not any study on this topic in Iran, this study aimed to determine the effects of a designed program on heart beat and oxygen saturation of infants. The special objectives were: 1) determining and comparing the mean of oxygen saturation of the infants before and during the deigned program in NICU, and 2) determining and comparing the NICU infants’ heart rate before and during the designed program.

## Methods

In a single group clinical trial study of before and after intervention 31 premature infants enrolled. The study population included all premature infants hospitalized in neonatal intensive care unit of the internal ward in Al-Zahra Hospital in Isfahan, who had the include criteria. The sample size calculated to be 31 infants with d = 3.3 and confidence coefficient of 95%. Sampling method was simple continued from May 1, 2008 to June 1, 2008.

Inclusion criteria were gestational age of 25-37 weeks, birth weight of 1000-2500 g, and being Iranian. Exclusion criteria included any change in infants’ care and treatment modality in the day of intervention, known mental, vision and hearing problems, known genetic disorders and known congenital abnormalities at birth, sepsis and gastro intestinal problems, infection, respiratory distress with respiratory score ≥ 5 and mother’s addiction.

Infants who needed phototherapy and those who underwent invasive procedures became sick and exposed to ventilator or pulse oximeter alarm during the intervention, were excluded from the study.

To collect demographic data, infants’ mothers were interviewed and also the newborn records were observed. Study variables included percentage of oxygen saturation and infants’ heart rate which was shown by monitors of pulse oximeter and were recorded in checklist by researchers and two nurses who were cooperating with them. The amount of light and sound in NICU were measured by luxmeter and sound meter. For the reliability of data collection instruments, the same devices were used during the study (YF-170 and CEL-18-56). To valid the instruments, these two devices were checked with another light meter and sound meter in Isfahan University of Medical Sciences. The researcher spoke to the authorities of Al-Zahra hospital and the head of NICU and explained the objectives of the study for them and also interviewed with parents to obtain their written consent for the study. To increase the accuracy of the study, at first a pilot study was conducted on 10 infants and after analyzing the results and conducting necessary changes in checklist for recording data, these 10 infants were excluded from the study. In the pilot study, the accuracy of observations by nurses was approved with a Cronbach’s alpha higher than 70%. At first, the researcher and her colleagues observed and recorded the situation for one hour between 12:00 to 13:00. During this hour, the amount of light and sounds in the NICU was assessed and recorded. Also, the heart rate of infants and their oxygen saturation were measured by the pulse oximeter and recorded every 5 minutes. The intervention was done from 14:30 to 15:30 of the same day. In this hour, the number of doctors, interns and students were less and therefore, it was easier for intervention. The researchers covered the infants’ room windows by curtains and reduced the light to about 10 lux, and for reducing sounds, asked the unit staff to lower down their voice, to minimize the phone ring sound, and the hospital matron was asked not to page during the intervention period. These strategies reduced the noises in NICU environment to level ≤45 dB. Also, during the intervention, infants’ moving was reduced to emergency situations. The infants’ heart beat rate and oxygen saturation were recorded every 5 minutes. Data were analyzed using SPSS and paired t-test.

## Results

Findings showed that out of 31 infants in the study 60% were boys and 35% were girls. Birth weights of most infants were first 1501 to 2000 g and secondly 1000 to 1500 g. Of all, 60% of study samples had 30-35 weeks of gestational age; and 65% were first born. At the time of intervention, most infants weighed 1000 to 1500 g. The duration of hospitalization in hospital was 1-5 days for 77% of infants. The age of infants at the time of intervention is presented in Table 1. From all, 72% of infants in the study were fed by their mothers’ milk and out of them, 41% were being breastfed method and 31% were using gavages method or oral by cup or syringes.

**Table 1:** Frequency distribution of infants’ age

Infant’s age	Number	Percentage
1-5 days	24	43.77
6-10 days	3	67.9
More than 10 days	4	9.12

Total	31	100

Regarding the first objective of the study, findings showed that the mean of oxygen saturation for infants at the time of intervention was significantly higher than the time of no intervention (p = 0.048) (Table 2).

**Table 2:** Comparing the means of oxygen saturation before and during the intervention

Index group	Minimum%	Maximum%	Mean%	SD	T	P
Before intervention	85.84	97.92	92.80	2.54	-2.56	0.048
During intervention	87.69	98.07	94.22	2.59		

Regarding the second objective, the results showed that the mean of heart rate in infants at the time of intervention was less than the time of no intervention, but the difference was not significant (Table 3).

**Table 3:** Comparing the means of infants’ heart rate before and during the intervention

Index group	Minimum/min	Maximum/min	Mean/min	SD	T	P
Before intervention	125.15	189.84	143.83	13.23	10.022	0.315
During intervention	117.00	182.76	136.84	13.76		

## Discussion

In the present study, most infants weighed between 1000 to 1500 g and it is the same as the study by Boo et al, in which the weight of infants was 1432 ± 226 g.^8^

Moreover, 72% of infants in the study were being breastfed, while in a study by Wooldridge et al, 60% of premature infants with the age of 30-35 weeks were being breastfed in the first week after birth.^9^ This difference can be related to the Iranian mothers’ more interest in breastfeeding compared to other societies.

The present study showed a significant difference between the mean of oxygen saturation of premature infants before and during intervention. This finding agrees with that of Anand’s study.^10^ In a study by Johnson et al in the US on infants’ responses to reducing noises in incubator, the results showed a significant relationship between the use of sound insulation foam inside the incubator to reduce noise and supportive treatment of oxygen therapy.^11^ The findings of the present study approves these results.

The above-mentioned studies approve that stimulants such as light and sound and also infants’ transfer are stress factors which can result in reducing arterial blood oxygen saturation. Also, Anand’s study that was published in 2000 found that infants’ stress lowers the blood oxygen saturation.^10^

The findings showed that infants’ heart rate during the intervention was lower than before, but the difference was not significant. Since stress increases heart beat by releasing catecholamines, decrease of heart beat is expected after the stress situation is corrected; the cause of insignificant difference in the present study can be related to the time (12:00 to 13:00) of the assessment of physiologic parameters before intervention. Perhaps the difference could be significant if these measurements were done before noon when the NICU was more crowded, or if the intervention was done for a longer time or with bigger size.

Brandon et al also said that sounds louder than 80 dB in NICU can increase the heart beat and decrease the arterial oxygen pressure.^12^

A study by Trapanotto also showed that NICU noises cause hospitalized infants’ behavioral and physiologic reactions and infants respond to stimulants specially loud sound showed significant behavioral changes.^13^

Since nurses have an effective role in reducing infants’ stress due to daily interventions and since considering the effect of visual and hearing stimulants is one of the complementary cares for premature infants to reduce their stress, and also because these two stimulants can interfere with growth of other sensory systems, the researchers hope that the findings of the present study can be used to improve safety of NICU environment. This measure may be followed by promotion of premature infants’ health.

At the end, it is recommended that interventions such as arranging the light of the environment and decreasing environment sounds be used as complementary medicine to improve premature infants’ health. Also, these interventions are recommended as major dimensions of care for infants hospitalized in NICU to improve their health.

The authors declare no conflict of interest in this study. Ethical committee approved the study.

## References

[1] Hockenberry MJ, Wilson D (2007). Wong’s nursing care of infants and children.

[2] Robert K, Behrman RE, Jenson HB, Stanton BF (2007). Nelson textbook of pediatrics.

[3] Jouybari L (2009). Nursing knowledge. Premature infants with inadequate weight measurement.

[4] Kenner C, Lott JW (2002). Comprehensive neonatal nursing: a physiologic perspective.

[5] Pillitteri A (2002). Maternal and child health nursing: care of the childbearing and childbearing family.

[6] Slevin M, Farrington N, Duffy G, Daly L, Murphy JF (2000). Altering the NICU and measuring infants’ responses. Acta Paediatr.

[7] Brandon DH, Holditch Davis D, Belyea M (2002). Preterm infants born at less than 31 weeks’ gestation have improved growth in cycled light compared with continuous near darkness. J Pediatr.

[8] Boo NY, Chee SC, Rohana J (2002). Randomized controlled study of the effects of different durations of light exposure on weight gain by preterm infants in a neonatal intensive care unit. Acta Paediatr.

[9] Wooldridge J, Hall WA (2003). Post hospitalization breastfeeding patterns of moderately preterm infants. J Prenat Neonatal Nurs.

[10] Anand KJ (2000). Effects of perinatal pain and stress. Prog Brain Res.

[11] Johnson AN (2003). Adapting the neonatal intensive care environment to decrease noise. J Perinat Neonatal Nurs.

[12] Brandon DH, Ryan DJ, Barnes AH (2007). Effect of environmental changes on noise in the NICU. Neonatal Netw.

[13] Trapanotto M, Benini F, Farina M, Gobber D, Magnavita V, Zacchello F (2004). Behavioral and physiological reactivity to noise in the new born. J Pediatr Child Health.

